# “We face the same risk as the other health workers”: Perceptions and experiences of community pharmacists in Indonesia during the COVID-19 pandemic

**DOI:** 10.1371/journal.pgph.0000606

**Published:** 2022-07-01

**Authors:** Luh Putu Lila Wulandari, Mishal Khan, Ari Probandari, Neha Batura, Astri Ferdiana, Yusuf Ari Mashuri, Tri Wibawa, Dea Daraninggar, Berlian Kusuma Dewi, Ric Day, Stephen Jan, Gill Schierhout, Shunmay Yeung, Virginia Wiseman, Marco Liverani

**Affiliations:** 1 The Kirby Institute, University of New South Wales, Sydney, Australia; 2 Faculty of Medicine, Universitas Udayana, Bali, Indonesia; 3 Department of Global Health and Development, London School of Hygiene & Tropical Medicine, London, United Kingdom; 4 Departments of Community Health Sciences & Pathology, Aga Khan University, Karachi, Pakistan; 5 Global Health Programme, Chatham House, London, United Kingdom; 6 Faculty of Medicine, Universitas Sebelas Maret, Surakarta, Indonesia; 7 Center for Tropical Medicine, Universitas Gadjah Mada, Yogyakarta, Indonesia; 8 Institute for Global Health, University College London, London, United Kingdom; 9 Faculty of Medicine, Universitas Mataram, Mataram, Indonesia; 10 Department of Microbiology, Faculty of Medicine, Public Health, and Nursing, Universitas Gadjah Mada, Yogyakarta, Indonesia; 11 St Vincent’s Clinical School, University of New South Wales, Sydney, Australia; 12 The George Institute for Global Health, Sydney, Australia; 13 Department of Clinical Research, London School of Hygiene & Tropical Medicine, London, United Kingdom; 14 School of Tropical Medicine and Global Health, Nagasaki University, Nagasaki, Japan; 15 Faculty of Public Health, Mahidol University, Bangkok, Thailand; Medicins Sans Frontiers - Operational Centre Brussels, London School of Hygiene & Tropical Medicine, LUXEMBOURG

## Abstract

In many countries, community pharmacies have played an important role during the COVID-19 pandemic, providing essential medicines and personal protective equipment (PPE), disseminating information on disease prevention and management, and referring clients to health facilities. In recognition of this, there are increasing calls for an improved understanding of the challenges and experiences faced by these providers during the COVID-19 pandemic, with a view to providing them with better support and guidance now and during future emergencies. Between January and February 2021 we conducted 21 qualitative interviews to explore the experiences, safety concerns, and attitudes of pharmacists and pharmacy technicians during the COVID-19 crisis in Indonesia, a country that has recorded more than four million cases since the start of the pandemic. Interview transcripts were analysed using thematic content analysis. Findings indicate that COVID-19 has had a significant impact on pharmacy practices in Indonesia. Most participants implemented preventive measures and adapted their business models to the changing circumstances. The shift to remote sales and home delivery allowed many pharmacies to maintain, and even increase their profit margins due to greater demand for medicines and PPE. However, many participants were concerned about the increased risk of infection due to limited social distancing and prolonged interactions with clients, many of whom displayed COVID-19 symptoms. Importantly, there was a general perception that the government did not sufficiently recognize these risks. In conclusion, the government should consider developing additional operational guidelines and regulatory frameworks to improve the safety, operation, and involvement of community pharmacies in the current pandemic response efforts and any future public health emergencies.

## Introduction

Community pharmacies are the first point of contact between clients and the health system in many countries, especially where the availability of public health services is limited [1]. As such, pharmacies across the world have played an important public health role during the COVID-19 pandemic, supplying essential medicines and personal protective equipment (PPE), conducting consultations for clients with mild symptoms and referring more serious cases, delivering information on disease prevention, contributing to COVID-19 surveillance, and ensuring the continuity of care delivery [2, 3]. In performing these activities however, community pharmacists may face a high risk of contracting the virus due to prolonged contact in the workplace with clients and colleagues, who may be infected [4]. Further, they can experience additional challenges due to lockdown measures and shortages of medicines and other supplies such as PPE [5, 6].

Aware of these challenges, professional organizations in the pharmacy sector have issued COVID-19 contingency plans to help their members during the current pandemics and any future outbreaks. In 2020, for example, the International Pharmaceutical Federation (FIP) published a detailed document containing relevant information and guidelines for the pharmacy workforce [7]. Similar documents were drafted by organisations at the regional level, such as the Federation of Asian Pharmaceutical Associations [8]. Guidelines were also developed at the national level by, for example, the National Health Service (NHS) in the United Kingdom [9] and by professional pharmacy organisations in Pakistan [2], Malaysia [10], South Africa [11], and the Philippines [12], to name a few. Despite these initiatives, however, several surveys have reported a general lack of access to information and training among pharmacists during the COVID-19 crisis [4, 13–16]. Furthermore, the experiences of pharmacists and pharmacy technicians during the pandemic have only been documented in a few studies. As a result, the generation of guidelines for pharmacists has tended to proceed without in-depth understanding of the needs and perspectives of these frontline workers.

Considering these issues, we conducted a qualitative study to explore the experiences and challenges of pharmacists and pharmacy technicians working during the COVID-19 outbreak in Indonesia, a country that has recorded more than four million cases and 150 thousand deaths since the start of the pandemic [17]. Besides the substantial health and economic burden, severe disruptions to health service delivery have been reported in Indonesia [18–20], including the closure of primary health centres (*puskesmas*) and hospitals in areas with widespread infection of local health workers [21]. In this context, community pharmacies have played an important public health role, serving their communities under challenging circumstances. Thus, gaining a better understanding of the challenges they have faced and coping strategies implemented is critical to inform national guidelines and prepare for future emergencies.

## Methods

### Study context

Indonesia has a large number of community pharmacies. According to recent estimates, there are around 130,000 licensed community pharmacies and drugstores serving a population of more than 270 million [22, 23]. Under the Indonesian legislation, community pharmacies can only run under the supervision of a pharmacist [24]. A pharmacist is a person with a bachelor’s degree in pharmacy and a registration training certificate [24, 25]. A pharmacy technician, on the other hand, is “a person who has graduated from a pharmacy assistant school, or has a three year diploma in pharmacy, or has a bachelor’s degree in pharmacy without holding a pharmacist registration training certificate” [25]. Unlike pharmacists, pharmacy technicians are not allowed to dispense medicines that require a prescription. They work closely with pharmacists to assist in the preparation and distribution of medicines to clients. Due to their popularity, community pharmacies are part of the *Jaminan Kesehatan Nasional* (JKN), the national health insurance scheme that was launched in 2014 to increase access to care and move the country towards universal health coverage. Under this scheme, Indonesian citizens are entitled to receiving care and essential medicines at public health facilities and participating private providers, including community pharmacies [26].

As in many other countries, community pharmacies in Indonesia have played an active role during the COVID-19 pandemic. In April 2020 the Indonesian government urged pharmacies to stay open and ensure access to medicines and essential services in the communities [27]. Furthermore, the Indonesian Pharmacists Association (*Ikatan Apoteker Indonesia or IAI*) produced standard operating procedures for their members, including advice on the use of PPE and sanitation, social distancing measures, and a case report form to refer suspected cases of COVID-19 to local hospitals for further medical examination and care [28].

### Research design

This qualitative study involved in-depth interviews with pharmacists and pharmacy technicians. A convenience sampling strategy was used [29]. Participants were identified in collaboration with the IAI and the Indonesian Pharmacy Technician Associations. An invitation letter to take part in the study and an information sheet summarising the purpose of the research were sent by the research team to all 34 provincial IAIs and the Indonesian Pharmacy Technician Associations in Indonesia, with 16 of them responding to the invitation. Each of these then sent the information to its members, with 21 indicating that they were interested in taking part in the study. The names and contact numbers of these potential participants were shared with the research team, who contacted them through the messaging application WhatsApp or email to obtain informed consent and schedule an interview.

### Data collection

Interviews were conducted between January and February 2021 using a semi-structured interview guide in Bahasa Indonesia, the main language of Indonesia. At that time, due to continued high levels of COVID-19 infections, the government extended its public activity restrictions, termed *Pemberlakuan Pembatasan Kegiatan Masyarakat (PPKM)*, in parts of Java and Bali including the closure of schools and work from home policy [30]. In line with the study objectives, the interviews were designed to explore: (1) sources of information about COVID-19; (2) perceptions about the impact of COVID-19 on pharmacy operations, including safety measures; and (3) views and attitudes towards the role of pharmacists in pandemic response efforts. The interview guide was piloted to refine the sequence and wording of questions and probes. Piloting was conducted with pharmacists who had a professional relationship with the research team in Indonesia. Summary notes were also taken about the interactions with each participant (including rapport and level of interest) to improve subsequent interviews and to determine data saturation. On average, interviews lasted 45 minutes and were recorded with only one exception, where consent was not given for recording the interview. Given social distancing measures, all interviews were conducted remotely using the Zoom teleconferencing platform, with cameras on to establish a personal connection between the researchers and the participants [31]. All interviews were conducted by three experienced local researchers, with training and experience in social research methods.

### Data analysis

Recorded interviews were transcribed verbatim, and processed by LPLW. The software NVivo 12 was used to aid thematic analysis. Inductive coding was used to identify patterns and emerging themes in the dataset. Five interview transcripts were translated into English for additional review by ML. The initial coding scheme was revised after iterative reading of the transcripts and discussion amongst the study team to reduce subjective bias in the qualitative data analysis. The Consolidated Criteria for Reporting Qualitative Research guidelines (COREQ) was used to ensure comprehensive reporting of the data collection and analysis procedures [32].

### Ethics approval

Ethics approval was granted by the Universitas Gadjah Mada (KE/FK/0464/EC/2020) and the University of New South Wales (HC191012) research ethics committees. Verbal informed consent was obtained for all in-depth interviews.

## Results

In the sections below, we present the findings structured around the main themes. Where appropriate, anonymised citations are included to illustrate key points and are referenced by the unique identifier IDI-*n*. In total we conducted 21 in-depth interviews with participants recruited from 16 provinces (Table 1). Most participants were pharmacists that had been working in the same establishment for at least three years. The age range of the study sample was 26–51 years. Apart from a pharmacy technician employed in a large retail chain, all participants worked in independent small pharmacies which have less than 11 staff.

**Table 1:** Characteristics of participants.

Code	Sex	Age	Province	Role	Type of establishment	Number of staff
IDI1	F	27	East Java	Pharmacist	Pharmacy	3
IDI2	F	33	Central Java	Pharmacy technician	Pharmacy (retail chain)	6
IDI3	F	38	Special District of Yogyakarta	Pharmacist	Pharmacy	6
IDI4	M	35	West Kalimantan	Pharmacist	Pharmacy	4
IDI5	M		West Java	Pharmacist	-	-
IDI6	M	33	Lampung	Pharmacist	Pharmacy	2
IDI7	F	51	Capital City of Jakarta	Pharmacist	Pharmacy	3
IDI8	F	26	Jambi	Pharmacist	Pharmacy	7
IDI9	M	41	East Java	Pharmacist	Pharmacy	6
IDI10	M	30	West Nusa Tenggara	Pharmacist	Pharmacy	5
IDI11	F	44	West Java	Pharmacist	Pharmacy	4
IDI12	F	40	Lampung	Pharmacist	Pharmacy	3
IDI13	F	32	South Sumatra	Pharmacist	Pharmacy	10
IDI14	M	43	North Kalimantan	Pharmacist	Pharmacy	4
IDI15	M	30	East Nusa Tenggara	Pharmacist	Pharmacy	4
IDI16	F	40	Riau	Pharmacist	Pharmacy	3
IDI17	F	31	Riau Island	Pharmacist	Pharmacy	3
IDI18	F	28	Bangka Belitung	Pharmacist	Pharmacy	5
IDI19	M	27	North Maluku	Pharmacist	Pharmacy	4
IDI20	M	32	Papua	Pharmacist	Pharmacy	1
IDI21	F	33	West Nusa Tenggara	Pharmacy technician	Drugstore	5

### Perceived adequacy and reliability of information about COVID-19

#### Information sources

Most participants were keen to receive constant updates about COVID-19 and reported a variety of information sources on the pandemic. The most cited information sources were the Ministry of Health, the District Health Office, and the IAI through their websites, webinars, and social media (IDI11, IDI13, IDI16). One pharmacist, who was a member of the South Sumatra committee of the IAI, said:

“This year I have attended dozens of webinars and teleconferences held by our organization and others… Overall there is a lot of information exchange among pharmacists, promoted by the Indonesian Pharmacists Association. And we can find many updates and training material on COVID-19”

(IDI13)

Notably, many participants stressed that social media platforms like Facebook, Instagram and, particularly, WhatsApp, were the most widely used and accessible sources of up-to-date information (IDI03, IDI04, IDI15, IDI18).

“The social media app we use the most is WhatsApp… because it’s easy”

(IDI10).

Some participants noted there was a lack of regular communication with the District Health Office regarding the safety of pharmacists. Further, despite the IAI having developed standard operating procedures, some participants mentioned that they were not sure whether they had received them.

#### Social media and false information

While social media provided easy access to updates on COVID-19, concerns emerged about the proliferation of false or inaccurate information through these channels:

“I am a member of professional [pharmacist organizations] and informal WhatsApp groups. In informal groups, family and friends often talk about COVID-19 and they say all sorts of things. As a pharmacist, I must warn them not to share false information (…) The situation will be chaotic if fake news is spread. We must counter that information”

(IDI10).

“During this pandemic, there are lots of hoaxes around. I often ask myself if this or that news is true, especially the information about vaccines. Vaccine hoaxes have gone crazy… they create panic. A couple of days ago, I even took part in a webinar on how to deal with hoaxes”

(IDI11)

### Impact of COVID-19 on pharmacy operations

#### “People are afraid to see the doctor… so they come here”

Some pharmacists said they had more clients than usual because people were worried about being forced into hospital quarantine if they were found to be infected with COVID-19 when visiting clinics and hospitals. For example, a pharmacist who was also working in a public health centre said:

“The number of clients who come to the health centre has decreased. Usually we have 100 or more clients [every day] but now we have much fewer clients, 30 or 50 at most. And when they come to my pharmacy, they often say they don’t want to go to the health centre or the hospital because they are afraid of being quarantined”

(IDI16)

Another participant said:

“People are afraid to see the doctor (…). They don’t go to the hospital because they don’t want to be detained if they test positive for COVID-19. So, they come here. I have many new clients (…), people who wouldn’t come here normally”

(IDI14)

#### Addressing shortages in PPE and medicines

Pharmacists reported a steep increase in demand for both medicines and PPE since the beginning of the pandemic. Products in high demand reportedly included antivirals (such as oseltamivir or favipiravir), vitamins (multivitamin complex, vitamin E, vitamin C), face masks, and antibiotics (such as azithromycin, amoxicillin, or cefadroxil). As participants noted, this has led to frequent stock outs (IDI05, IDI011, IDI012, IDI016, IDI019, IDI020):

“People pile face masks up. Because of this, we can easily get to the point when face masks are no longer available. And that’s when they will sell at a very high price. In fact, I couldn’t get a face mask for 3 months, I think, maybe more (…). No face masks and hand sanitizer”

(IDI19)

“A few months ago, face masks were suddenly gone… they were really rare, hard to find”

(IDI20)

In this context, participants implemented different strategies to manage shortages of medicines and PPE, including sourcing from different wholesalers, producing hand sanitizers themselves, purchasing scarce products from other pharmacies, or using alternative brands. In addition, some pharmacies refused to sell products in bulk to individuals whom they had reason to believe wanted to resell them:

“We sell face masks in large quantities only to hospitals. Other people can still buy face masks from us, but only for personal use. And yet many people come here to buy one or two large packs for resale. We must say no to them”

(IDI07)

“I live in [X], an industrial area, so many people come here to buy large quantities [of medicines or face masks], saying they are for their employees in the factories. But if the client doesn’t bring a proof to support this claim, such as a signed letter, we won’t sell them. Because we know that bulk purchase is often for resale, and this leads to shortages”

(IDI11)

#### Perceived increase in the price of medicines and PPE

Two participants felt that high demand had pushed up the prices of many products, especially vitamins and PPE.

“Ester-C [vitamin supplement] used to be cheap. It was around 35 or 45 thousand rupiahs [USD 2.45–3.15] per unit. With COVID-19, however, the price has continued to increase. Now we buy Ester-C at 90 thousand and we sell it at 100 thousand rupiah. If buy large quantities from whole sellers though, we can get lower prices”

(IDI11)

“One box of face masks normally costs about 30 thousand [rupiahs]… last year it soared to 80 thousand, then 150 thousand, then 250 thousand, and then 450 thousand. This is a really high price”

(IDI05)

#### Remote sales and services

Similar to other sectors, social distancing measures and changing consumer behaviour due to health concerns forced many pharmacists in Indonesia to provide remote services through telephone orders, online sales and home delivery. For those who were able to adapt, this proved to be a successful business strategy:

“Today I haven’t sold much in the pharmacy… but I made lots of transactions online. My clients order a medicine online or by phone. After I receive the order, I send them the parcel with xxx [a within-city delivery parcel shipping company]. Much of our work is online in this period and people can receive their medicines easily”

(IDI11)

In pursuing online sales, some pharmacists relied on e-commerce services:

“We can sell our products using xxx [the e-commerce services]. This helps us. So, the increase in sales is actually from online sales”

(IDI01)

#### Safety concerns

Participants were aware of the risk of infection with COVID-19 in the workplace and therefore, implemented a range of protective measures. As highlighted in the quotes below, it was common for pharmacy staff to wear a face mask or face shield, keep a safe distance from clients, install physical barriers, and use hand sanitizer after every interaction with clients:

“We are always vigilant. We wear masks and the clients cannot be too close. We stay at least one meter away from the clients”

(IDI12)

“We take many precautions. During counseling, for example, we keep much more distance from the client. This is a bit inconvenient”

(IDI19)

“We provide vitamins to our employees. In addition, we use a protective barrier to limit our interactions with the clients”

(IDI09)

Despite these measures, some participants were anxious about clients coming to their pharmacies with symptoms of COVID-19 or asking for medications perceived to be related to COVID-19 such as vitamins, antivirals, and antibiotics. In addition, frequent shortages of masks heightened the concerns of pharmacy staff:

“I mean there is scarcity of masks just outside the city where I live. Because of this [pharmacy staff] often don’t wear masks, so the risk [of infection] in the pharmacy is very high (…) With lack of self-protection, my colleagues working there have a much higher risk”

(IDI01)

### Involvement in COVID-19 response efforts and professional duties

#### Advising clients about the management of COVID-19

Pharmacists in our sample were proud to support the pandemic response and said they would often remind their clients about the main preventative measures (the ‘5 Ms’) outlined in the government’s health and safety protocol including: the wearing of face masks; social distancing; hand washing; crowd avoidance; and mobility restrictions. A few participants also mentioned they had displayed banners to further promote these measures:

“We often explain to our clients they need to follow the protocol and avoid crowds. That’s what we do”

(IDI10)

“We are pharmacists, so we have a responsibility for increasing awareness of the 3Ms policy, which is now 5Ms”

(IDI9)

“We cannot organise large health education meetings in the communities because it would be too risky. But we can do other things. Since the start of the pandemic, we have displayed banners to remind our clients of prevention measures and how to wash hands properly”

(IDI18)

One pharmacist also mentioned that she would often provide advice by telephone about home remedies and medications for colleagues and friends who were recovering from COVID-19:

“I support my friends in self-isolation. I know a lot of health workers… and I know a lot of cases among them. So, for example I am assisting the family of a nurse who got COVID-19”

(IDI11)

#### Complaints about the lack of sufficient recognition by the government

While most pharmacists were happy to be involved in the pandemic response, one felt that the government did not sufficiently recognize their contributions and risks, particularly in comparison with hospital-based pharmacists.

“Some people think that our risk is not as high as that faced by our colleagues in the hospitals or clinics, who must work in close contact with sick people [with COVID-19]. So, people believe that those who can come to the pharmacy are still strong and healthy. That is what makes the pharmacy’s risks being perceived as not as high as those at a hospital, clinic, or the health centre. But this is not true–we face the same risk of COVID-19 as the other health workers”

(IDI1)

Similarly, another said that stronger regulations to support community pharmacists are difficult to implement due to their limited lobbying power, compared to pharmacists working in the hospital setting:

“The voice of the community pharmacists is rarely heard in Indonesia. What is often heard is the voice of the hospital pharmacists because they are closer to the government. For example, hospital pharmacists organise frequent meetings with national or international stakeholders where they can talk to them… But the situation of community pharmacists is much different. We are not really involved in national or international activities”

(IDI14)

The same participant went on to explain that a clear regulatory framework was needed to guide their actions and expanded roles now and for future pandemics.

“The field of clinical pharmacy is well developed in Indonesia. So, pharmacists could contribute much more to the pandemic response. Imagine, for example, a client with COVID -19… If the prognosis is good and the pharmacist is allowed to advise on treatment, many clients could be provided with home care without having to burden the hospitals. That would be really helpful, also considering there are so many pharmacists in Indonesia… …. But pharmacists cannot help much because the legal bases are weak…”

(IDI14)

## Discussion

Most participants in our sample coped well with the unprecedented challenges posed by COVID-19, implementing preventive measures and adapting their business model to the changing circumstances. The shift to remote sales and home delivery allowed many pharmacies to continue operating, and even increase their sales due to greater demand for medicines and PPE.

Nonetheless, significant operational challenges emerged. Despite preventive measures implemented in pharmacies, many participants were concerned about the increased risk of infection due to prolonged interactions with clients, many of whom were suspected to be contagious. Importantly, as documented in other countries [4], there was a general perception that these risks were not sufficiently recognized by the government.

Frequent stock outs of face masks, hand sanitizers, and medicines were reported in many pharmacies. While this has been a global challenge also affecting doctors and nurses [33], it has been particularly acute in low- and middle-income countries, like Indonesia, where supply chains are more vulnerable to fluctuations in the availability of imported products and raw materials [34]. Similar to findings in Serbia [5] and Ethiopia [35], some pharmacists were worried about the lack of face masks for their personal use and safety. Participants also noted that these supply disruptions led to steep price increases especially for vitamins and face masks, as found in other countries [6, 36]. Pharmacists in our study reported frequent stockouts of vitamins. This is likely to have been driven by advice from the Indonesian government to take multivitamin supplements to help strengthen immune system response to COVID-19 (21).

Our findings invite some reflections for policy, practice, and research. Our study clearly indicates that measures to ensure the safety of pharmacy personnel must be strengthened. While pharmacists in our sample had access to training materials and updates on COVID-19 from professional organizations, government guidelines and safety protocols targeting community pharmacies were not widely circulated. Second, frequent stock outs of key products in some pharmacies highlights the need for constant monitoring of sales data to avoid supply shortages and price increases [37]. Additionally, monitoring the sales of particular products such as vitamins, cough medicines, antivirals and antipyretics could also serve to support the national system of syndromic surveillance [38], as seen in other countries [39]. To achieve this goal, effective oversight of the supply chain and regulations should be in place. In the United States, for instance, the Food and Drug Administration adopted a flexible approach to allow for the local production of face masks during the pandemic [40]. Alternatively, the government of Taiwan applied restrictions on the quantity face masks which could be purchased by individual citizens during the pandemic [41]. Governments can also mandate greater public disclosure of prices for pharmaceutical products in high demand during pandemics, as was the case in Rwanda [6].

Moreover, there is little doubt that the increase in online sales has helped sustain supplies of products and consultation services amid the pandemic. However, this practice is not without its challenges. Concerns have been raised in other countries about potential hazards associated with online sales of medicine, with little or no oversight from the authorities [42]. Thus, a stronger regulatory framework and some forms of quality control should be introduced. In some European countries, for example, the following information must be included in the parcel together with the medicines purchased online: information on the pharmacy, a notice that medicines cannot be sent back (except if defective), the list of pharmaceutical care services offered after dispensing and any information required for their responsible use [43]. On a related note, our study also suggests that the pandemic resulted in a steep increase in the demand for antibiotics, despite these medicines not being recommended for the management of COVID-19. Given the harmful consequences of this practice on antimicrobial resistance [44], the government should consider implementing additional measures to monitor antibiotic use and promote antibiotic stewardship during the pandemic. This is in line with wider calls to better integrate private providers including community pharmacies into the health systems of LMIC in order to tackle ongoing threats such as antimicrobial resistance [45].

Taken together, our findings suggest that during the COVID-19 pandemic, community pharmacies in Indonesia have fulfilled an important public health role, supporting the continued supply of medicines and services, advising clients on disease prevention and management, promoting preventive measures, and even countering the proliferation of false or inaccurate information from social media. This is in keeping with a recent review, which highlighted the potential for ‘multilevel’ engagement of pharmacists during the COVID-19 pandemic, from disease education and counselling to active surveillance of suspicious cases [46]. And yet, similar to findings in Pakistan [47], some participants noted that more guidance and a clearer regulatory framework are needed to support pharmacies and enable them to contribute to pandemic response efforts. Indeed, limited coordination and recognition of the important role of the private sector, including pharmacies, has been a long-standing challenge to effective public health action in many countries, and has been a major shortcoming of pandemic preparedness in the past [48] and during the COVID-19 crisis [3, 49–51]. In a study in Puerto Rico, for example, pharmacists were resentful and outraged they were excluded from all government schemes aimed at recognizing and incentivizing essential workers and health professionals, despite providing continued service since the start of the COVID-19 pandemic [3]. In contrast, evidence suggests that countries that have engaged with the private sector, through for example, purchasing arrangements or the rapid adaptation of existing policies around the management of drug supplies [37], have been able to implement more effective COVID-19 response measures [52]. For example, during the COVID-19 pandemic, the government of Iran established a public-private partnership with the country’s biggest online retailer, Digikala, to promote the sale of products in high demand during the pandemic through their platform, free of charge [53]. Other countries have issued novel legal provisions to extend the role of pharmacists during the pandemic including authorisation to: prepare disinfectants; renew prescriptions for chronic diseases; fill *pro auctore* and *pro familia* prescriptions (i.e. for themselves and for some family members); perform COVID-19, influenza and Group A *Streptococcus* screening tests; and administer vaccine [54]—actions that are worth considering in the Indonesian setting during the current pandemic and for future public health crises.

### Study limitations

This study was conducted with limited resources and in difficult circumstances due to the lockdown measures that were imposed in Indonesia to control the pandemic at the time of data collection. As a result, we could focus only on licensed pharmacies and we could not capture the views and experiences of informal drug outlets, which are also widespread and a popular source of medicines and PPE in Indonesia. Other important actors in the pharmacy sector, including suppliers, local and central authorities, and representatives of pharmaceutical companies were also not interviewed as part of this study. In addition, the reliance on professional pharmacists’ organizations to recruit participants may have introduced sampling bias. Despite ensuring anonymity and confidentiality, some interviews may have also been affected by social desirability bias, which occurs when respondents say something that is considered socially acceptable or in line with social norms [55].

## Conclusion

COVID-19 has increased the recognition of the critical role that community pharmacies play in the health system. Our study provides novel insights on the views and experiences of these frontline health care providers during the pandemic. While some community pharmacies have managed to maintain, and even increase, their services using e-commerce and other remote sale strategies, several key challenges to maintaining quality service provision have emerged. While pharmacists were exposed to an abundance of information on COVID-19 through various online sources, concerns emerged about the circulation of inaccurate information through these channels. Frequent stockouts and a steep increase in the price of medicines and other supplies also arose as key challenges. Lastly, participants expressed that their contributions and vulnerabilities during the COVID-19 pandemic have not been sufficiently recognized by the government. Thus, further attention to these issues should be given in future research and policy in order to better understand the role of the pharmacy sector during public health emergencies. A more comprehensive study involving all categories of stakeholders (including informal drug outlets, suppliers, and representatives of professional organisations) would be a useful follow-up to this study, providing key evidence to inform operational guidelines and regulatory frameworks for pandemic preparedness.

## Supporting information

S1 File Interview guide. (DOCX)

S2 File COREQ guidelines for qualitative research. (DOCX)
