# Supplementary material for: “We face the same risk as the other health workers”: Perceptions and experiences of community pharmacists in Indonesia during the COVID-19 pandemic
Source: PLOS Glob Public Health. 2022 Jul 1;2(7):e0000606. doi: 10.1371/journal.pgph.0000606 (PMC10021738; doi:10.1371/journal.pgph.0000606)
Supplement: S1 File — (DOCX) [file pgph.0000606.s001.docx]

**Interview Guide**

**Instructions for the interviewer:** *Introduce yourself, then explain the participant the background and the aim of the interview.*

*After informed consent has been obtained, remind the respondent that they have no obligations to answer any questions. In addition, they can stop the interview at any time and if they decide to do so there will be no consequences for them. They should also be encouraged to take their time and if they cannot answer a question right away, they can return to it later.*

**Opening**

Please can you tell me a little a bit about yourself and where you work? [PROBE]: how long have been working there and who else works with you?

What do you know about the COVID situation in your area?

Type of PDS

Number of employees

location

**Knowledge and awareness of COVID-19**

Some people working at pharmacies believe they don’t have sufficient understanding of COVID-19. What are your thoughts?

**Probe:**

What are your main sources of information about COVID-19?

Guidelines?

Resources?

Do you think this is enough?

[IF THIS IS NOT ENOUGH]:

- What specific things would you like more information about?
- And where would you like to find this additional information? (eg social media, tv, radio, websites)
- Media they often use?

**Impact of COVID-19 on work routines and business management**

Do you feel COVID-19 had had a big impact on your work in the pharmacy (or the drug store)? [IF SO], can you explain?

**Probe:**

Are there more or less customers? Do they come with the same or different problems?

Some pharmacists said there is a huge problem with stockouts due to COVID-19. Did you experience any shortages in COVID-related products (such as masks, hand sanitizer, etc)?

Did you experience shortages in other products or medicines? [IF SO], can you explain?

[IF APPROPRIATE]: Did you do anything to mitigate these problems? [IF SO], can you explain?

Have you felt at risk of getting COVID yourself? Anything that could be done to make it safer for you?

**The role of pharmacists in the response to COVID-19**

Have you ever suspected that some of your clients had COVID-19? [IF SO]: Why? What kind of symptoms? What did you do?

In general, do you think pharmacists in Indonesia currently play an active role in the response to COVID-19? Can you explain?

Interview/consultation?

Selling products?

Key informants?

What role do you think pharmacies should play in the COVID-19 response?

**Probe:**

Do you think they should do more? [IF SO]: What should they do and how?

Do you think you need more resources to do these things? [PROBE]: What kind of resources?

Roles of online pharmacies?

Do their websites offer specific services?

Their roles in comparison to before pandemic?

**Closure**

Anything else you would like to discuss? Any other challenges?

Thank you so much for your time. If you would like to know more about our project, you can access the project website at [: [PINTAR STUDY](https://www.pintarstudy.org/)](https://www.pintarstudy.org/)

On the website, you can also find contact information in case you have any questions or you would like to check on the study progress and results.
