# Supplementary material for: “We face the same risk as the other health workers”: Perceptions and experiences of community pharmacists in Indonesia during the COVID-19 pandemic
Source: PLOS Glob Public Health. 2022 Jul 1;2(7):e0000606. doi: 10.1371/journal.pgph.0000606 (PMC10021738; doi:10.1371/journal.pgph.0000606)
Supplement: S2 File — (DOCX) [file pgph.0000606.s002.docx]

**COREQ guidelines for qualitative research**

**Title “We face the same risk as the other health workers”: perceptions and experiences of community pharmacists in Indonesia during the COVID-19 pandemic**

| **No** | **Item** | **Description** | **Response** |
| --- | --- | --- | --- |
| **Domain 1: Research team and reflexivity** | | | |
| Personal characteristics | | | |
| 1. | Interviewer/facilitator | Which author/s conducted the interviews or focus groups? | LPLW, AF, YAM |
| 2. | Credentials | What were the researcher's credentials? E.g. PhD, MD | AF is a medical doctor with a PhD in public health from the University of Groningen. LPLW is a medical doctor with a PhD in public health from the University of New South Wales. YM is a medical doctor. Three of them are experienced qualitative researchers. ML and MK are associate professors of health policy and systems at the London School of Hygiene and Tropical Medicine. AP is a Professor in Public Health at Universitas Sebelas Maret in Indonesia. |
| 3. | Occupation | What was their occupation at the time of the study? |  |
| 4. | Gender | Was the researcher male or female? | AF, AP and LPLW are female. ML and YM are male. |
| 5. | Experience and training | What experience or training did the researcher have? | ML and MK are social scientists with extensive experience in qualitative methods. LPLW, AP, and AF are public health researchers and have been involved in many qualitative research projects and publications |
| Relationship with participants | | | |
| 6. | Relationship established | Was a relationship established prior to study commencement? | Researchers were not familiar with participants prior to the data collection. |
| 7. | Participant knowledge of the interviewer | What did the participants know about the researcher? e.g. personal goals, reasons for doing the research | The researchers introduced themselves to all participants, providing information about their work and employment.  In the informed consent form and prior to the interview, all participants were informed about study aims |
| 8. | Interviewer characteristics | What characteristics were reported about the interviewer/facilitator? e.g. Bias, assumptions, reasons and interests in the research topic |  |
| **Domain 2. Study design** | | | |
| Theoretical Framework | | | |
| 9. | Methodological orientation and theory | What methodological orientation was stated to underpin the study? e.g. grounded theory, discourse analysis, ethnography, phenomenology, content analysis | Thematic analysis was used by performing inductive coding to identify patterns and emerging themes in the dataset. |
| 10. | Sampling | How were participants selected? e.g. purposive, convenience, consecutive, snowball | A convenience sampling strategy was used. (p. 7) |
| 11. | Method of approach | How were participants approached? e.g. face-to-face, telephone, mail, email | The names and contact numbers of these potential participants were shared with the research team, who contacted them through the messaging application WhatsApp or email to obtain informed consent and schedule an interview(p. 7) |
| 12. | Sample size | How many participants were in the study? | 21 in total (p. 10) |
| 13. | Non-participation | How many people refused to participate or dropped out? Reasons? | None of the potential participants refused to participate in the study or dropped out during the interview |
| 14. | Setting of data collection | Where was the data collected? e.g. home, clinic, workplace | Interviews were conducted using the software Zoom (p. 9) |
| 15. | Presence of non-participants | Was anyone else present besides the participants and researchers? | No. |
| 16. | Description of the sample | What are the important characteristics of the sample? | The characteristics of participants are summarised in the Methods section (p. 10) |
| Data collection | | | |
| 17. | Interview guide | Were questions, prompts, guides provided by the authors? Was it pilot tested? | The interviews were pilot tested. Details of the interview approach are provided at p. 8 |
| 18. | Repeat interviews | Were repeat interviews carried out? If yes, how many? | No |
| 19. | Audio/visual recording | Did the research use audio or visual recording to collect the data? | All interviews, except one, were audio recorded (p. 8) |
| 20. | Field notes | Were field notes made during and/or after the interview or focus group? | Yes, (p. 8) |
| 21. | Duration | What was the duration of the interviews or focus group? | The average duration was about 45 min (p. 9) |
| 22. | Data saturation | Was data saturation discussed? | Yes, see notes at p. 8 |
| 23. | Transcripts returned | Were transcripts returned to participants for comment and/or correction? | No., ashis would have been logistically laborius. |
| **Domain 3. Analysis and findings** | | | |
| 24. | Number of data coders | How many coders coded the data? | One researcher: LPLW. However, findings and emerging themes were repeatedly discussed with ML and all research team members. (p. 9) |
| 25. | Description of the coding tree | Did authors provide a description of the coding tree? | Themes were derived from the data using an inductive process (p. 9) |
| 26. | Were themes identified in advance or derived from the data? | Were themes identified in advance or derived from the data? |  |
| 27. | Software | What software, if applicable, was used to manage the data? | QSR NVivo version 12 (p. 9) |
| 28. | Participant checking | Did participants provide feedback on the findings? | No, as this would have been logistically laborious |
| Reporting | | | |
| 29. | Quotations presented | Were participant quotations presented to illustrate the themes / findings? Was each quotation identified? e.g. participant number | Yes, pleas see Results section |
| 30. | Data and findings consistent | Was there consistency between the data presented and the findings? | Yes |
| 31. | Clarity of major themes | Were major themes clearly presented in the findings? | Yes (see Results section) |
| 32. | Clarity of minor themes | Is there a description of diverse cases or discussion of minor themes? | Yes |
